# Soluble SIRP-Alpha Promotes Murine Acute Lung Injury Through Suppressing Macrophage Phagocytosis

**DOI:** 10.3389/fimmu.2022.865579

**Published:** 2022-05-12

**Authors:** Qinjun Shen, Li Zhao, Linyue Pan, Dandan Li, Gang Chen, Zhihong Chen, Zhilong Jiang

**Affiliations:** ^1^ Department of Pulmonary Medicine, Zhongshan Hospital, Fudan University, Shanghai, China; ^2^ Department of Pathology, Zhongshan Hospital, Fudan University, Shanghai, China; ^3^ Department of Pulmonary and Critical Care Medicine, Henan Provincial People’s Hospital, People’s Hospital of Zhengzhou University, Zhengzhou, China

**Keywords:** SIRP-alpha, acute lung injury, macrophages, phagocytosis, STAT6, STAT3

## Abstract

Soluble signal regulatory protein-alpha (SIRP-alpha) is elevated in bronchoalveolar lavage (BAL) of mice with lipopolysaccharides (LPS)-induced acute lung injury (ALI). To define the role of soluble SIRP-alpha in the pathogenesis of ALI, we established murine ALI in wild-type (WT) and SIRP-alpha knock-out (KO) mice by intratracheal administration of LPS. The results indicated that lack of SIRP-alpha significantly reduced the pathogenesis of ALI, in association with attenuated lung inflammation, infiltration of neutrophils and expression of pro-inflammatory cytokines in mice. In addition, lack of SIRP-alpha reduced the expression of pro-inflammatory cytokines in LPS-treated bone marrow-derived macrophages (BMDMs) from KO mice, accompanied with improved macrophage phagocytosis. Blockade of soluble SIRP-alpha activity in ALI BAL by anti-SIRP-alpha antibody (aSIRP) effectively reduced the expression of TNF-alpha and IL-6 mRNA transcripts and proteins, improved macrophage phagocytosis *in vitro*. In addition, lack of SIRP-alpha reduced activation of Src homology 2 domain-containing protein tyrosine phosphatase 1 (SHP-1) and improved activation of signal transducer and activator of transcription-3 (STAT3) and STAT6. Suppression of SHP-1 activity by tyrosine phosphatase inhibitor 1 (TPI-1) increased activation of STAT3 and STAT6, and improved macrophage phagocytosis, that was effectively reversed by STAT3 and STAT6 inhibitors. Thereby, SIRP-alpha suppressed macrophage phagocytosis through activation of SHP-1, subsequently inhibiting downstream STAT3 and STAT6 signaling. Lack of SIRP-alpha attenuated murine ALI possibly through increasing phagocytosis, and improving STAT3 and STAT6 signaling in macrophages. SIRP-alpha would be promising biomarker and molecular target in the treatment of murine ALI and patients with acute respiratory distress syndrome (ARDS).

## Introduction

Signal regulatory protein-alpha (SIRP-alpha) is a transmembrane glycoprotein and pre-dominantly expressed in myeloid and neural cells (1). After activation of SIRP-alpha, Src homology 2 domain-containing protein tyrosine phosphatase-1 (SHP-1) is recruited and regulates receptor tyrosine kinase-coupled signaling, participating in the regulation of phagocytosis and polarization of macrophages (2). Recent studies revealed that SIRP-alpha is up-regulated by granulocyte-macrophage colony-stimulating factor (GM-CSF) and suppressed by extracellular signal-regulated kinase (ERK) inhibitor and glucocorticoids (3). Thus, glucocorticoids may exert anti-inflammatory role by suppressing SIRP-alpha, subsequently improving macrophage phagocytosis, clearance of dead cells and tissue debris in inflamed local tissues. Surfactant protein D (SP-D) (4, 5) and CD47 (6–9) are important ligands of SIRP-alpha, participating in suppression of macrophage phagocytosis. Because the potent role of CD47/SIRP-alpha signaling in suppression of macrophage phagocytosis, blocking the CD47/SIRP-alpha interaction between macrophages and tumor cells has become a promising approach in cancer immunotherapy (10–12).

However, the role of SIRP-alpha in inflammatory diseases, particularly in murine acute lung injury (ALI) and patients with acute respiratory distress syndrome (ARDS) is not well investigated so far. Previous reports showed that SIRP-alpha suppressed macrophage activation, reduced the expression of IL-12 and induced graft tolerance (13). Therefore, SIRP-alpha is considered as an anti-inflammatory receptor on macrophages. Intact SP-D and SP-A are thought to exert anti-inflammatory function through binding to SIRP-alpha on macrophages (2, 14). Rho-associated protein kinase (ROCK), ERK1/2, p38alpha/beta mitogen-activated protein kinase (p38alpha/beta MAPK) and p38gamma/delta MAPK signaling pathways are possibly involved in the suppressive effects (3, 15). In contrast to the anti-inflammatory function of SIRP-alpha *in vitro*, recent studies in animal models have revealed that SIRP-alpha had pro-inflammatory function and increased inflammation-mediated insulin resistance (16, 17). In addition, it is evidenced that lack of SIRP-alpha provided protective effects on mice with ischemia reperfusion-induced acute kidney injury, in association with reduced expression of proinflammatory cytokines and mediators, such as reactive oxygen species (ROS), thrombospondin-1 (TSP-1) and CD47 (18). TSP-1 and CD47 exerted pro-inflammatory function in some animal models by binding to SIRP-alpha, such as renal ischemia reperfusion injury (19). Lack of CD47 expression and blockade of CD47 activity by CD47-Fc and CD47 fusion protein (CD47-Var1) can effectively reduce SIRP-alpha+ dendritic cell trafficking into mediastinal lymph node, suppressing Th2 type immune responses in asthma and the expression of IL-1beta and TNF-alpha in patients with Crohn’s disease (20–22). Therefore, blocking TSP-1/SIRP-alpha and CD47/SIRP-alpha interaction would be useful therapeutic approach in the treatment of some inflammatory diseases. However, it is not defined so far whether SIRP-alpha exerts the pro-inflammatory function in these animal models through affecting macrophage phagocytosis.

Previous report indicated that different forms of SIRP-alpha protein may account for distinct roles of SIRP-alpha in modulation of immune responses. Membrane-bound SIRP-alpha can be proteolytically cleaved and released during inflammation through a disintegrin and metalloproteinase domain-containing protein 10 (ADAM10). However, the role of soluble SIRP-alpha is not well studied so far. It was recently reported that overexpression of proteolytically cleaved SIRP-alpha fragments enhanced activation of STAT-1 (Signal transducer and activator of transcription) and NF-kappaB pathway *in vitro* (2). However, it is not well defined the role and underlying molecular mechanisms of cell membrane-bound and soluble form of SIRP-alpha in the development of ALI/ARDS.

In this study, we for the first time found that soluble SIRP-alpha was highly elevated in bronchoalveolar lavage (BAL) of murine ALI. To further define the role of soluble SIRP-alpha in the pathogenesis of ALI/ARDS, we established murine ALI in WT and SIRP-alpha knock-out (KO) mice. The results revealed that lack of SIRP-alpha significantly reduced the severity of murine ALI, in association with reduced production of pro-inflammatory cytokines and improved macrophage phagocytosis through STAT3 and STAT6 signaling pathways.

## Material and Methods

### Mice and Treatment

SIRP-alpha^+/-^ mice on C57BL/6 background were created by Cyagen biotech company in Suzhou, China, using Crispr/Cas9 technique, in which exons 7 and 8 that encode majority of the cytoplasmic region were deleted. SIRP-alpha^-/-^ KO mice were obtained by mating of SIRP-alpha^+/-^ females bred to SIRP-alpha^+/-^ male. Mouse phenotypes were identified by Terra PCR direct genotyping kit (San Jose, CA) and flow cytometry analysis. PCR primer sequences for PCR genotyping were listed in **Table 1**.

**Table 1:** Primer sequences for genotyping.

F1: 5'-TCATTCCAGCTTCATCAGGAGGGAG-3' R1: 5'-TAGCAGTTCCATGAGGACATAAGAC-3' F2: 5'-ACTGCTCTTGGGTGACCTGAATG-3'	

8-10 weeks old of male WT and SIRP-alpha KO mice were intratracheal (i.t.) injected with 5 mg/kg lipopolysaccharides (LPS) (Sigma-Aldrich, St Louis, MO) for 2 days. The mice treated with PBS were used as controls. BAL and lung tissues were collected for analysis. All animals were housed and treated under the guidelines of the Institutional Animal Care and Use Committee of the Fudan University Zhongshan Hospital in China. All experiments were approved by the committee and performed in the Zhongshan Hospital Fudan University.

### Hematoxylin and Eosin (H&E) Staining and Pathological Quantification of Lung Sections

Paraffin-embedded lung tissue sections were deparaffinized in xylene and hydrated by passing through decreasing concentration of alcohol baths (100%, 90%, 80%, 70%) and water. The slides were then stained in hematoxylin for 3-5 minutes and dipped in 1% acid alcohol (1% HCl in 70% alcohol) for 30 sec after washed with tap water. The slides were then counterstained with 1% Eosin Y for 10 min and followed by dehydration, clearing in two xylene baths, finally mounted in mounting media. The pathological score of lung tissues was semi-quantified under a light photomicroscope by double-blind method. The severity of lung injury was evaluated by scale from 0 to 4 in terms of alveolar edema, hemorrhage, alveolar septal thickening, and infiltration of polymorphonuclear leukocytes.

### Flow Cytometry Analysis

0.5×10^6^ cell suspension of lung tissue digests, BAL and cultured cells were incubated with antibody cocktail containing FITC-anti-CD80, PerC-Cy5-anti-F4/80, PE-Cy7-anti-Ly6G, APC-anti-CD11b (BioLegend. San Diego, CA), APC-Cy7-anti-SIRP-alpha, BV421-anti-Siglec-F (BD Biosciences, Franklin Lakes, NJ and eBioscience, San Diego, CA) for 40 minutes in dark. Cells stained with fluorescence minus one (FMO) were used as controls. The stained cells were analyzed on BD FACSAria III flow cytometer. All data were analyzed by Flow Jo software, version 8.8.4 (Becton, Dickinson and Company, Franklin Lakes, NJ).

### Western Blot Analysis

Total protein concentration was measured by the BCA Assay. 5 µg cell-free BAL protein or 20 µg macrophage protein lysates per lane were denatured in reducing Laemmli buffer, then separated by 10% SDS-PAGE gel electrophoresis and transferred onto nitrocellulose membranes. Glyceraldehyde 3-phosphate dehydrogenase (GAPDH) was used as an internal loading control for cell protein lysate samples, but not used in cell-free BAL samples, because GAPDH content in cell-free BAL was changed in response to lung inflammation, according to the previous reports (23–25). The blots were then incubated with rat anti-human SIRP-alpha (1:500 dilution) for 2 h, followed by incubation with horseradish peroxidase (HRP)-conjugated anti-rat IgG for 1 h. After washing with TBST buffer, the blots were developed by enhanced chemiluminescence (ECL) substrate solution (Amersham Biosciences, Piscataway, NJ, USA). Band densitometric intensity was quantitatively analyzed by ImageJ software.

### Culture and Treatment of BMDMs

Bone marrow cells were collected by flushing femurs and tibiae with PBS and maintained in RPMI1640 medium supplied with 10% fetal bovine serum (FBS) and 20% conditional media of NIH3T3 cells for 6 days to obtain bone marrow-derived macrophages (BMDMs). WT and SIRP-alpha KO BMDMs were stimulated with 500 ng/ml LPS or 30% BAL from naïve and ALI mice (naïve BAL, ALI BAL). The untreated or naïve BAL-treated cells were used as controls. The treated cells and supernatants were analyzed for protein expression and mRNA transcripts.

### Immunostaining Assay

The treated BMDMs were fixed with 4% paraformaldehyde for 10 min and blocked with blocking buffer (10% goat serum and 0.05% Triton X-100 in PBS) for 30 min. The cells were then incubated with indicated primary antibody (dilution 1:200) for 24 hours at 4°C and Cy3-conjugated secondary antibody (dilution 1:400) at room temperature for 2 hours. The positively stained cells were visualized under fluorescence microscope with 200 × magnification. Primary antibodies included anti-total SHP-1, anti-p-SHP-1, p-STAT6 (Signal transducer and activator of transcription 6) and acetyl-STAT3 (Abcam, Cambridge, MA and Cell signaling technology, Danvers, MA), anti-mouse TNF-alpha and IL-6 (R&D systems, Minneapolis, MN). The positively stained cells were quantitatively analyzed on Image J software and data was presented as ratio of arbitrary units to controls.

### ELISA Assay

The concentration of TNF-alpha, IL-6, CXCL15 and IL-18 in BAL and cell supernatants were measured by ELISA assay according to industrial instructions (R&D systems). Soluble SIRP-alpha in naïve BAL and ALI BAL was measured by direct ELISA assay. Briefly, BAL was diluted in coating buffer (dilution 1:3) and coated in 96-well Maxisorp plate, then incubated subsequently with rat anti-SIRP-alpha antibody (dilution 1:500) and HRP-conjugated anti-rat IgG (dilution 1:1000). Data was presented as ratio of OD450nm value to PBS control.

### qRT-PCR Assay

RANTES and IL-6 mRNA transcripts in the treated BMDMs were analyzed by qRT-PCR analysis, as previously reported (24). Briefly, total RNA was extracted from the treated cells by TRIzol reagent (Invitrogen, Grand Island, NY) and cDNA was synthesized by ReverTra Ace qPCR RT kit. Quantitative PCR was performed using SYBR green real-time PCR Master Mix (Toyobo, Osaka, Japan). Mouse TNF-alpha and IL-6 primers were synthesized by Shanghai BioSune Biotechnology, according to the sequences previously reported (24). GAPDH was used as internal control. Real-time PCR reaction was performed on QuantStudio 5 real-time PCR systems (Applied biosystems) under condition of 95°C for 2 min, 40 cycles (95°C for 30 s, 57°C for 30 s, and 72°C for 40s). Gene expression was calculated by formula of 2^-ΔΔCt^. Data was presented as mean ΔΔCt relative to GAPDH ± standard deviation.

### Macrophage Phagocytosis Assay

WT and SIRP-alpha KO BMDMs were pre-treated with 5 μM C188-9 (STAT3 inhibitor), 5 μM AS1517499 (STAT6 inhibitor), 5 μM TPI-1 (tyrosine phosphatase inhibitor 1, SHP-1 inhibitor) respectively for 30 min, and followed by treatment with or without 500 ng/m LPS, 30% naïve BAL or ALI BAL, respectively. PKH26 fluorescent cell linker (Sigma, Saint Louis, Missouri)-labeled apoptotic neutrophils (NPs) were added at a ratio of 2:1 (apoptotic cells: macrophages) for 4 hours. NPs were obtained from mouse bone marrow and purified on 65-75% Percoll gradient (GE Healthcare). Neutrophil apoptosis was induced by exposure to germicidal UV light source for 15 min and incubated at 37°C for 12 hrs. All inhibitors were purchased from Selleckchem, Houston, TX. Macrophage phagocytosis was analyzed by flow cytometry and visualized under fluorescence microscope. Data was presented as mean percentage of PKH26+ cells after gating on CD11b+ cells.

### Statistical Analysis

Results are presented as mean ± standard deviation of each group. All data were statistically analyzed by using GraphPad Prism 7 software. Student’s *t* test was performed for comparison between two groups and one-way analysis of variance (ANOVA) followed by Tukey’s multiple comparisons test was performed for comparison over two groups. A value of *p*<0.05 was considered as statistically significantly different.

## Results

### SIRP-Alpha Was Highly Expressed in Mice With LPS-Induced ALI

**Figure 1:**
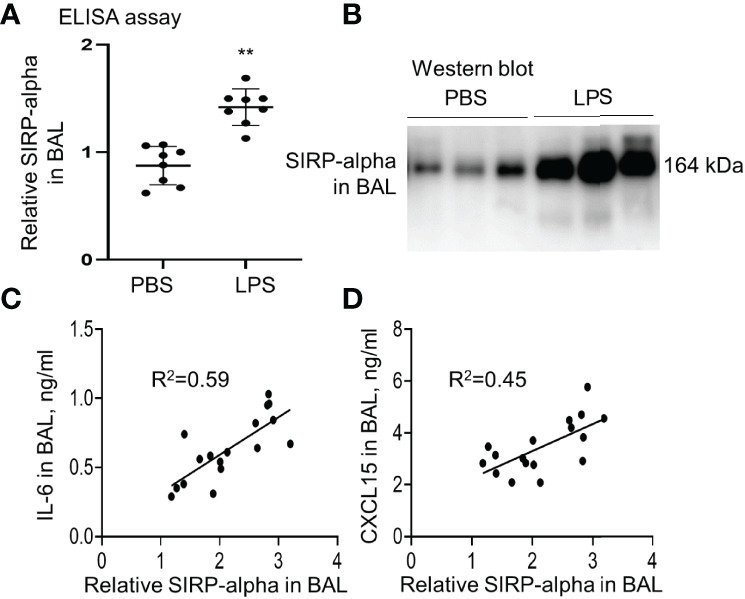
The expression level of SIRP-alpha was increased in BAL of mice with LPS-induced acute lung injury (ALI). **(A)** Direct ELISA assay for soluble SIRP-alpha in BAL of mice with LPS-induced ALI or treated with PBS control. Data was presented as ratio of OD450nm value to PBS control, **p < 0.01 vs. PBS group. 2-tailed student *t* tests. **(B)** Western blot analysis for soluble SIRP-alpha protein in BAL. One representative blot. Each lane represents individual sample of each mouse. **(C, D)** Correlation analysis between the expression levels of soluble SIRP-alpha and IL-6 or CXCL15 in BAL. Each point presents the value of individual sample.

Our study in LPS-induced murine ALI revealed that soluble SIRP-alpha protein was significantly elevated in BAL of mice with ALI (**Figures 1A, B**). However, membrane-bound SIRP-alpha protein on cell surface was not significantly elevated (data not shown). In addition, the increased soluble SIRP-alpha in BAL was positively correlated to the expression levels of pro-inflammatory cytokine IL-6 and chemokine CXCL15 in ALI BAL (**Figures 1C, D**). Thereby, soluble SIRP-alpha in BAL would be a novel biomarker and possibly involved in the development of murine ALI.

### Lack of SIRP-Alpha Reduced Lung Inflammation in SIRP-Alpha KO Mice With LPS-Induced ALI

**Figure 2:**
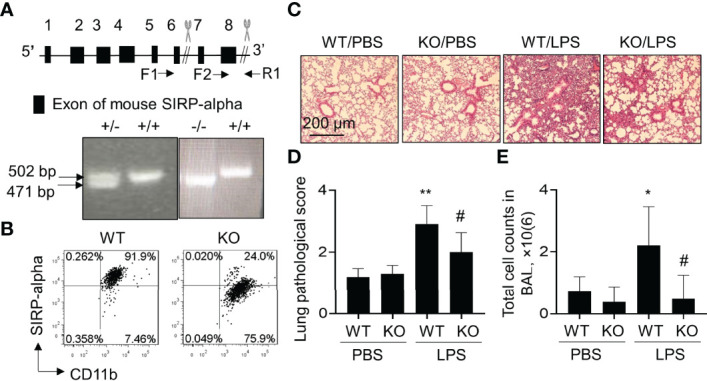
Blockade of SIRP-alpha expression suppressed the development of murine ALI. **(A)** Schematic diagram and genotyping of SIRP-alpha phenotypes in mice by PCR. 502 bp: SIRP-alpha ^+/+^ (WT); 471 bp: SIRP-alpha ^-/-^ (KO); 502/471 bp: SIRP-alpha ^+/-^. **(B)** Flow cytometry analysis of SIRP-alpha expression on CD11b+ myeloid cells in BAL of naïve WT and KO mice. Representative dot plot. **(C)** H&E staining for lung pathology. Mice with ALI were established by intratracheal injection of 5 mg/kg LPS for 2 days. Mice treated with PBS were controls. Representative photograph of each treatment with 100 × magnification. **(D)** Quantitative analysis of lung pathology by H&E staining. The score of severity was evaluated by scale from 0 to 4 in terms of alveolar edema, hemorrhage, alveolar septal thickening and infiltration of polymorphonuclear leukocytes. **(E)** Total cell counts in BAL. Data was presented as mean ± standard deviation, *p < 0.05, **p < 0.01 vs. PBS group; ^#^p < 0.05 vs. WT group, n=5-6. One-way ANOVA followed by Tukey’s multiple comparisons test.

To further investigate the role of SIPR-alpha in the development of ALI, we established a conventional SIPR-alpha KO mouse model. The heterogenous and homologous SIRP-alpha deficient mice were identified by PCR method. As a result, we observed 471 bp and 502 bp PCR products, respectively by primer pairs F1/R1 and F2/R1. 471 bp products represented defect and 502 bp PCR products presented intact SIPR-alpha genes, respectively (**Figure 2A**). SIRP-alpha expression on myeloid cell surface was further analyzed by flow cytometry analysis, in which SIRP-alpha protein expression was largely reduced in CD11b+ BAL cells of naïve SIRP-alpha KO mice, compared to that in WT mice (**Figure 2B**).

To further investigate the effects of SIRP-alpha deficiency on the development of ALI, we intratracheal (i.t.) treated WT and SIRP-alpha KO mice with 5 mg/kg LPS for 2 days. BAL and lung tissues were collected for analysis. The results showed that LPS treatment significantly increased acute lung parenchyma inflammation, exudation of fluid and destruction of normal tight alveolar endothelial–epithelial barrier in WT mice. However, lack of SIRP-alpha expression in KO mice significantly attenuated LPS-induced acute lung inflammation and tissue injury (**Figures 2C, D**). Consistently, total cell counts in BAL of KO mice were significantly reduced, compared to those in WT mice (**Figure 2E**). Therefore, lack of SIRP-alpha effectively protected mice from the development of murine ALI.

### Lack of SIRP-Alpha Suppressed Infiltration of Neutrophils and Expression of Pro-Inflammatory Cytokines in Murine ALI

**Figure 3:**
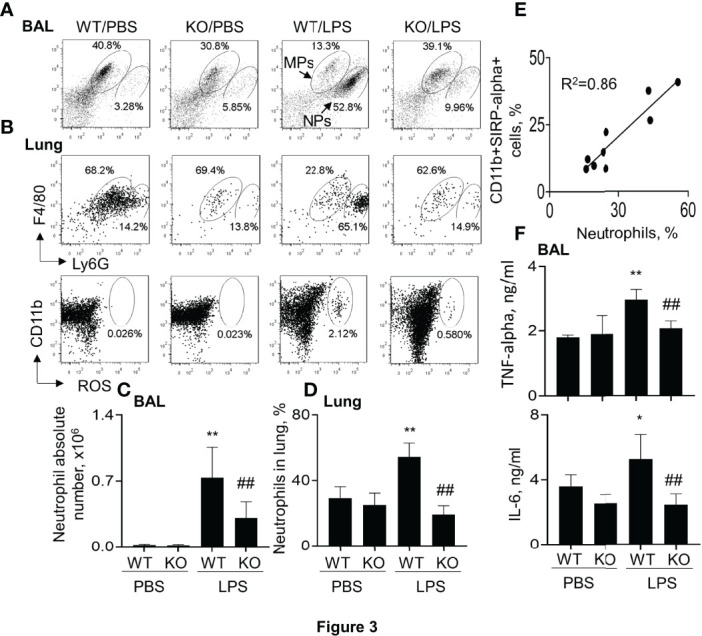
Lack of SIRP-alpha reduced neutrophil infiltration and expression of pro-inflammatory cytokines in murine ALI. **(A)** Flow cytometry analysis for the infiltrating neutrophils (NPs) and macrophages (MPs) in BAL (A) and lung digests (B, upper panel). NPs were identified as F4/80(low)Ly6G(high) cells; MPs were identified as F4/80 (high) Ly6G (low) cells. Cell mitochondria-derived reactive oxygen species (ROS) was analyzed by respiratory burst assay kit **(B)**, lower panel). Representative dot plot. **(C, D)** Quantitative analysis of neutrophil absolute number in BAL and the percentage of NPs in lung tissues. **(E)** Correlation analysis between the percentage of NPs and SIRP-alpha expression in the lung digests. Each point presents individual sample. **(F)** ELISA analysis for the expression of TNF-alpha and IL-6 in BAL. All quantitative data was presented as mean ± standard deviation, *p < 0.05, **p < 0.01 vs. PBS group; ^##^ p < 0.01 vs. WT group, n=5-6. One-way ANOVA followed by Tukey’s multiple comparisons test.

Further analysis in the murine ALI model indicated that the percentage and absolute number of F4/80+Ly6G+ neutrophils (NPs) were significantly increased in BAL and lung digests of WT mice. However, lack of SIRP-alpha significantly decreased the percentage and absolute number of NPs in KO mice, compared to those in WT mice with ALI (**Figures 3A–D**). In addition, we observed that the percentage of NPs and CD11b+SIRP-alpha+ cells was positively correlated in the lung digests (**Figure 3E**), indicating the involvement of SIRP-alpha in the induction of neutrophil infiltration into lung tissues of ALI (**Figure 3E**). In addition, we found that lack of SIRP-alpha expression reduced production of reactive oxygen species (ROS), an important oxidative molecule (26) in the lung tissues of KO mice, compared to that in WT mice (**Figure 3B**, lower panel). The results were consistent with the expression levels of cytokines, in which pro-inflammatory cytokines, TNF-alpha and IL-6 were significantly reduced in the lung tissues of KO mice, compared to those in WT mice (**Figure 3F**). Thus, lack of SIRP-alpha protected mice from the development of ALI, in association with reduced infiltration of neutrophils and expression of pro-inflammatory cytokines.

### Siglec-F(-) Subtype Macrophages and Neutrophils Were Reduced in SIRP-Alpha Deficient Mice With ALI

**Figure 4:**
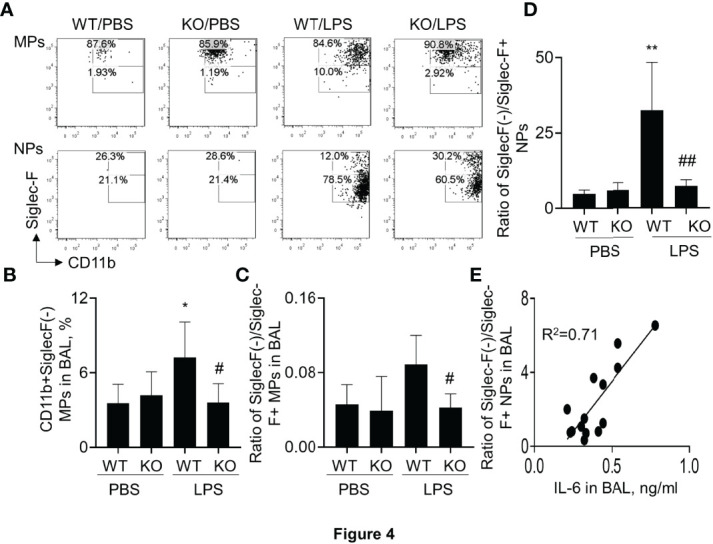
Lack of SIRP-alpha expression attenuated CD11b+Siglec-F(-) subtype macrophages and neutrophils in murine ALI. **(A)** Flow cytometry analysis for CD11b+Siglec-F(-) subtype MPs and NPs in BAL of murine ALI. CD11b+Siglec-F(-) subtype MPs and NPs were gated on MPs and NPs, respectively. Representative dot plot. **(B)** Quantitative analysis of CD11b+Siglec-F(-) subtype MPs in BAL. **(C, D)** Ratio of CD11b+Siglec-F(-) subtype MPs/CD11b+Siglec-F+ subtype of MPs and NPs in BAL. Data was presented as mean ± standard deviation, *p<0.05, **p<0.01 vs. PBS group; #p<0.05, ## p<0.01 vs. WT group for **(B–D)**. n=5-6. One-way ANOVA followed by Tukey’s multiple comparisons test. **(E)** Correlation analysis between the expression of IL-6 in BAL and ratio of Siglec-F(-) subtype/Siglec-F+ subtype NPs in BAL. Each point presents individual sample.

It was previously reported that Siglec-F was highly expressed in M2-like macrophages (25, 27). To further investigate the effects of SIRP-alpha on Siglec-F expression, the Siglec-F+ and Siglec-F(-) subtype cells were measured by flow cytometry after gating on macrophages and neutrophils. The results showed that the percentage of Siglec-F(-) subtype macrophages and neutrophils were effectively increased in macrophages and neutrophils of WT mice with ALI. However, their percentage was significantly attenuated in KO mice, compared to those in WT mice (**Figures 4A, B**). Consistently, the ratio of SiglecF(-)/Siglec-F+ subtypes of macrophages and neutrophils was significantly reduced in BAL of KO mice, compared to that in WT mice (**Figures 4C, D**). In addition, the ratio of SiglecF(-)/Siglec-F+ subtype neutrophils was positively correlated to the expression level of IL-6 in BAL (**Figure 4E**). Therefore, SiglecF(-) subtype macrophages and neutrophils would have a pro-inflammatory property in murine ALI and that would be considered as a novel cell biomarker in murine ALI.

### Lack of SIRP-Alpha Expression in Macrophages Reduced the Expression of Pro-Inflammatory Cytokines and Improved Macrophage Phagocytosis *In Vitro*

**Figure 5:**
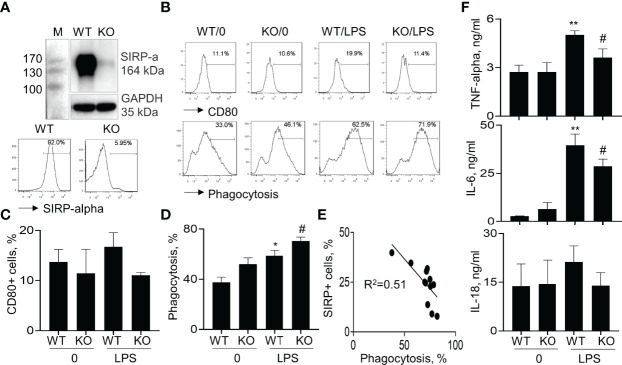
Lack of SIRP-alpha expression suppressed activation, expression of pro-inflammatory cytokines and improved phagocytosis of bone marrow-derived macrophages (BMDMs). **(A)** Western blot (upper panel) and flow cytometry (lower panel) analysis for the expression of SIRP-alpha in WT and KO mice-derived BMDMs. Representative blot and histogram of flow cytometry. **(B)** BMDMs from WT and KO mice were treated with or without 500 ng/ml LPS for 24 hours. Flow cytometry analysis for the expression of CD80 and macrophage phagocytosis of PKH26-labeled apoptotic NPs at a ratio of 2:1 (apoptotic cells: macrophages) for 4 hours. Representative histogram. **(C, D)** Quantitative analysis for CD80+ cells and phagocytosis activity of macrophages. Phagocytosis activity was presented as the percentage of PKH26+ cells after gated on CD11b+ cells. **(E)** Correlation between macrophage phagocytosis and the percentage of SIRP-alpha+ cells. Each point presents individual sample. **(F)** ELISA assay for the expression of TNF-alpha, IL-6 and IL-18 in the supernatants of treated cells. All quantitative data was presented as mean ± standard deviation. *p < 0.05, **p < 0.01 vs. 0 group, ^#^p < 0.05 vs. WT group, n=3. One-way ANOVA followed by Tukey’s multiple comparisons test.

To further confirm that lack of SIRP-alpha expression attenuated ALI through suppressing macrophage activation and expression of pro-inflammatory cytokines *in vitro*, we treated BMDMs from WT and KO mice with or without LPS, respectively. Flow cytometry analysis and Western blot analysis of cell samples showed that SIRP-alpha expression was significantly reduced in KO BMDMs, compared to that in WT BMDMs, confirming SIRP-alpha deficiency in KO BMDMs (**Figure 5A**). In addition, we observed that lack of SIRP-alpha moderately reduced LPS-induced expression of CD80, indicating the role of SIRP-alpha in inducing activation of macrophages (**Figure 5B** upper panel, and **Figure 5C**). In addition, we observed the increased macrophage phagocytosis of apoptotic neutrophils after LPS treatment, that was further improved by SIRP-alpha deficiency in macrophages (**Figure 5B** lower panel and **Figure 5D**). Furthermore, the macrophage phagocytosis activity was negatively correlated to the expression level of SIRP-alpha in macrophages (**Figure 5E**), indicating the suppressive effects of SIRP-alpha on macrophage phagocytosis *in vitro*. Consistent with the reduced macrophage activation in KO BMDMs, we observed the lower expression levels of TNF-alpha, IL-6 and IL-18 in the cell supernatants of LPS-treated KO BMDMs, compared to those in LPS-treated WT BMDMs (**Figure 5F**). The results confirmed the role of SIRP-alpha in promoting pro-inflammatory responses and suppressing macrophages phagocytosis.

### Blockade of SIRP-Alpha Activity on Macrophages by SIRP-Alpha Neutralizing Antibody Reduced the Expression of Pro-Inflammatory Cytokines and Improved Macrophage Phagocytosis

**Figure 6:**
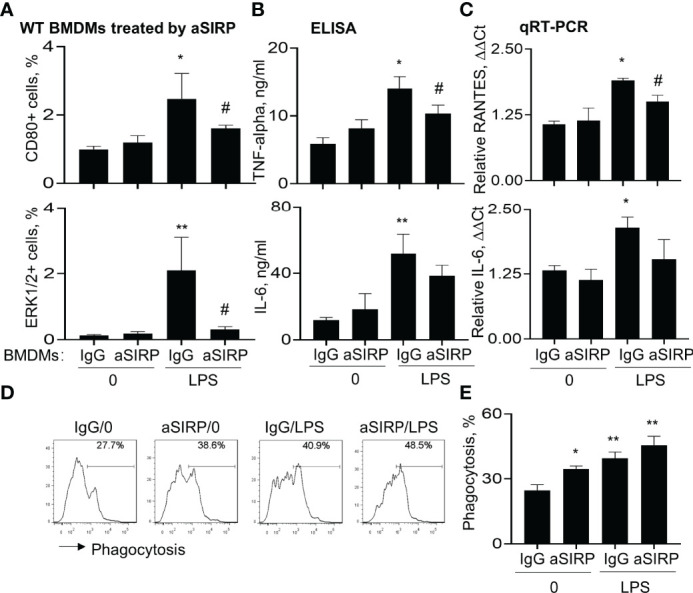
Blockade of SIRP-alpha on macrophages by neutralizing antibody suppressed activation, expression of pro-inflammatory cytokines and improved macrophage phagocytosis. WT BMDM cells were pre-treated with 2 μg/ml anti-SIRP-alpha (aSIRP) or IgG isotype control for 1 hours and followed by stimulation with or without 500 ng/ml LPS for 24 hours. **(A)** Quantitative analysis of CD80+ and ERK1/2+ cells after flow cytometry. **(B)** ELISA analysis for the expression levels of TNF-alpha and IL-6 in the supernatants of treated cells. **(C)** mRNA transcripts of RANTES and IL-6 in the lysates of treated cells were measured by qRT-PCR. Data was presented as ΔΔCt. **(D)** Phagocytosis activity of the treated macrophage was measured by addition of PKH26-labeled apoptotic NPs at a ratio of 2:1 (apoptotic cells: macrophages) for 4 hours. Representative histogram. **(E)** Quantitative analysis of macrophage phagocytosis after treatment. All quantitative data was presented as mean ± standard deviation. *p < 0.05, **p < 0.01 vs. 0 group, ^#^p < 0.05 vs. IgG group, n=3. One-way ANOVA followed by Tukey’s multiple comparisons test.

The role of SIRP-alpha in macrophage pro-inflammatory cytokine expression and phagocytosis were further confirmed in BMDMs pre-treated with anti-SIRP-alpha neutralizing antibody (aSIRP). Consistently, blockade of SIRP-alpha activity by aSIRP effectively reduced LPS-induced activation of macrophages. We observed that CD80 and ERK1/2 positive cells were increased by LPS treatment, that was effectively reversed by aSIRP pre-treatment (**Figure 6A**). In addition, ELISA analysis of cell supernatants and qRT-PCR analysis of the treated cells revealed lower expression levels of TNF-alpha, IL-6 and RANTES in aSIRP pre-treated WT BMDMs than those in the untreated WT BMDMs (**Figures 6B, C**). However, the suppressive effects of aSIRP on these cytokine expressions were not observed in KO BMDMs that lack SIRP-alpha expression on cell surface, indicating the requirement of cell membrane-bound SIRP-alpha in modulation of macrophages (data not shown). Consistently with the results in **Figure 5D**, we observed the additive effects of aSIRP and LPS in improving macrophage phagocytosis of apoptotic neutrophils (**Figures 6D, E**).

### Blockade of Soluble SIRP-Alpha in BAL Reduced the Expression of Pro-Inflammatory Cytokines and Improved Phagocytosis in Macrophages

**Figure 7:**
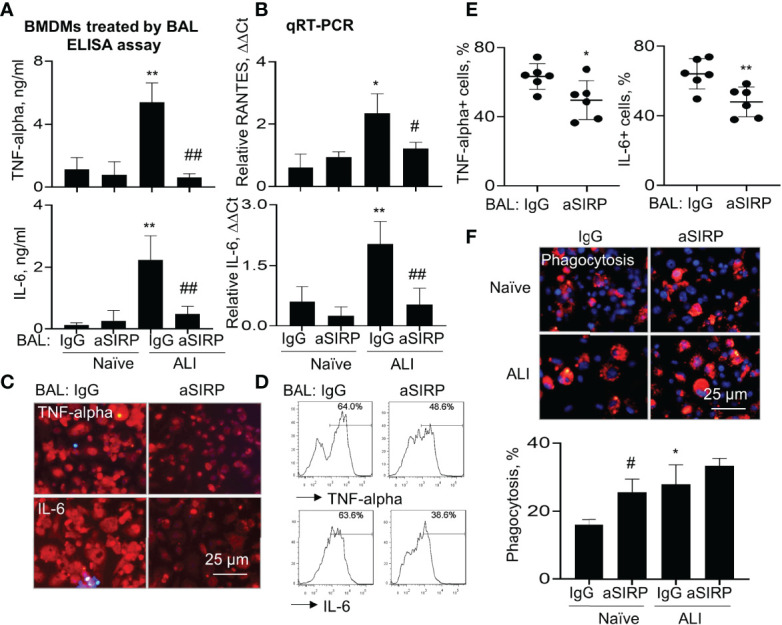
Blockade of soluble SIRP-alpha in BAL by neutralizing antibody suppressed activation, expression of pro-inflammatory cytokines and improved macrophage phagocytosis. WT BMDMs were treated with 30% ALI BAL that was pre-neutralized with 2 μg/ml aSIRP or IgG isotype. **(A)** The expression levels of TNF-alpha and IL-6 in the supernatants of 24 hours-treated cells were measured by ELISA assay. **(B)** RANTES and IL-6 mRNA transcripts in the lysates of treated cells were measured by qRT-PCR. Data was presented as mean ΔΔCt ± standard deviation. *p < 0.05, **p < 0.01 vs. naive BAL group, ^#^p < 0.05, ^##^p < 0.01 vs. IgG group, n=3. One-way ANOVA followed by Tukey’s multiple comparisons test. **(C, D)** The expression of TNF-alpha and IL-6 in ALI BAL-treated cells was analyzed by Immunostaining (representative photograph, 200 × magnification) and flow cytometry (representative histogram). **(E)** Quantitative analysis of TNF-alpha and IL-6 positive cells after flow cytometry. *p < 0.05, **p < 0.01 vs. IgG group, 2-tailed student’s *t* test. **(F)** Macrophage phagocytosis of PKH26-labeled (red) apoptotic NPs after treatment with naïve and ALI BAL (representative photograph, 200 × magnification, upper panel). Blue: DAPI-stained nuclei. Quantitative analysis of macrophage phagocytosis (lower panel). Data was presented as mean percentage of PKH26+ cells ± standard deviation, * p < 0.05 vs. naïve BAL; ^#^ p < 0.05 vs. IgG, n=3. One-way ANOVA followed by Tukey’s multiple comparisons test.

To further determine the role of BAL-derived soluble SIRP-alpha in macrophage activation and phagocytosis, we collected naïve BAL and ALI BAL from naïve mice and ALI mice, then pre-neutralized the soluble SIRP-alpha protein in naïve and ALI BAL with aSIRP or IgG isotype control. The treated BAL were then added into WT BMDMs for 24 hours. ELISA analysis of cytokines in macrophage supernatants indicated that there were higher expression levels of TNF-alpha and IL-6 in ALI BAL-treated cells than those in naïve BAL-treated cells. However, pre-neutralizing soluble SIRP-alpha in ALI BAL significantly reduced the expression of these cytokines in the treated cell supernatants, compared to the IgG pre-neutralized controls (**Figure 7A**). In addition, we observed the lower levels of TNF-alpha and IL-6 in the supernatants of BAL-treated BMDMs than those in BAL-untreated BMDMs, as shown in **Figure 6B**, that may be caused by addition of 30% BAL into BMDMs, resulting in dilution of cell culture medium, suppression of cell growth and production of pro-inflammatory cytokines. Considering the supernatants of BAL-treated cells contained both BAL-derived and macrophage *de novo* expressed TNF-alpha and IL-6, we further analyzed the cytokine levels in the treated cells by qRT-PCR, immunostaining and flow cytometry analysis. As a result, we observed that soluble SIRP-alpha in ALI BAL effectively induced the expression of IL-6 and RANTES transcripts in the treated cells, that was effectively reversed by aSIRP pre-treatment (**Figure 7B**), consistent with the results of immunostaining (**Figure 7C**) and flow cytometry (**Figures 7D, E**), in which the expression of TNF-alpha and IL-6 was obviously reduced in BMDMs treated with aSIRP pre-neutralized ALI BAL, compared to the cells treated with IgG-treated controls. However, macrophage phagocytosis was moderately improved in BMDMs treated with aSIRP pre-neutralized BAL (**Figure 7F**). The studies *in vitro* provided solid evidence that soluble SIRP-alpha in ALI BAL activated macrophages and induced the expression of pro-inflammatory cytokines, and suppressed macrophage phagocytosis.

### Lack of SIRP-Alpha Increased Macrophage Phagocytosis Through STAT3 and STAT6 Signaling Pathways

**Figure 8:**
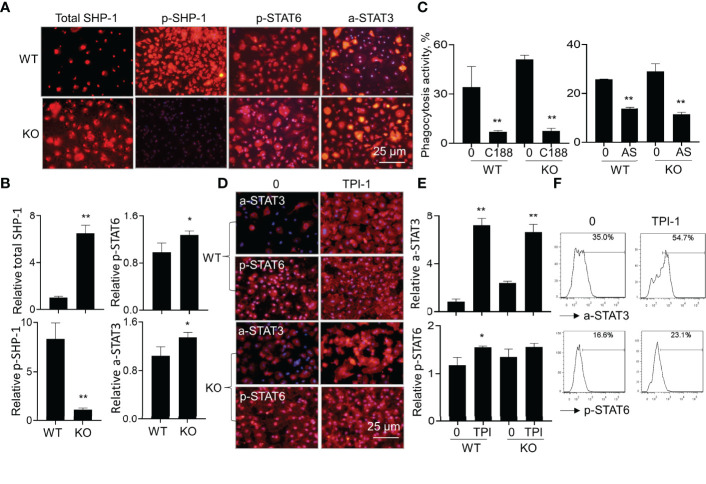
Lack of SIRP-alpha improved macrophage phagocytosis through STAT3 and STAT6 signaling pathways. **(A)** Immunostaining for total SHP-1, phosphorylated SHP-1 (p-SHP-1), phosphorylated STAT6 (p-STAT6) and acetylated STAT3 (a-STAT3) in WT and KO BMDMs 12 hours after LPS treatment. Red: positively stained cells. Representative photograph, 200 × magnification. **(B)** Quantitative analysis for total SHP-1, p-SHP-1, p-STAT6 and a-STAT3 by ImageJ software. Data was presented as mean relative fluorescence intensity over WT BMDMs ± standard deviation, *p < 0.05, **p < 0.01 vs. WT group, n=3, 2-tailed student’s *t* test. **(C)** Phagocytosis activity of WT or KO BMDMs treated by 5 μM C188-9 (STAT3 inhibitor) or 5 μM AS1517499 (STAT6 inhibitor). The untreated cells were controls. Data was presented as mean ± standard deviation, **p < 0.01 vs. untreated cells, n=3. **(D)** Immunostaining for a-STAT3 and p-STAT6 in WT or KO BMDMs after treatment with 5 µM TPI-1 (SHP-1 inhibitor) or untreated. Red: positively stained cells, representative photograph, 200 × magnification. **(E)** Quantitative analysis of a-STAT3 and p-STAT6 in TPI-1 treated WT or KO BMDMs by ImageJ software. Data was presented as mean relative fluorescence intensity over untreated WT BMDMs ± standard deviation, *p < 0.05, **p < 0.01 vs. untreated cells, n=3. One-way ANOVA followed by Tukey’s multiple comparisons test. **(F)** Flow cytometry analysis for a-STAT3 and p-STAT6 in the untreated or TPI-1 treated WT BMDMs. Representative histogram.

To investigate downstream signaling pathways of SIRP-alpha in modulation of macrophages, we analyzed the expression of total SHP-1, phosphorylated SHP-1 (p-SHP-1), phosphorylated STAT6 (p-STAT6) and acetylated STAT3 (a-STAT3) in WT and KO BMDMs by immunostaining. The results showed that lack of SIRP-alpha induced more expression of total SHP-1, and largely diminished activation of SHP-1, compared to that in WT BMDMs, indicating the requirement of SIRP-alpha in the activation of SHP-1. In addition, we observed more p-STAT6 at residue Tyr641 (p-STAT6) and acetyl-STAT3 at residue Lys685 (a-STAT3) in KO BMDMs than those in WT BMDMs, indicating the suppressive effects of SIRP-alpha on the activation of STAT3 and STAT6 (**Figures 8A, B**). To further investigate the role of STAT3 and STAT6 signaling in macrophage phagocytosis, we pre-treated BMDMs with STAT3 inhibitor, C188-9 and STAT6 inhibitor, AS1517499 respectively. The results showed that blockade of STAT3 and STAT6 activation by STAT3 and STAT6 inhibitors significantly reduced macrophage phagocytosis in both WT and KO BMDMs (**Figure 8C**). The results indicated that STAT3 and STAT6 signaling were at downstream of SIRP-alpha and promoted macrophage phagocytosis. Therefore, lack of SIRP-alpha expression or blockade of SIRP-alpha activity by aSIRP improved macrophage phagocytosis and suppressed production of pro-inflammatory mediators possibly through increasing STAT3 and STAT6 signaling pathways in macrophages.

**Figure 9:**
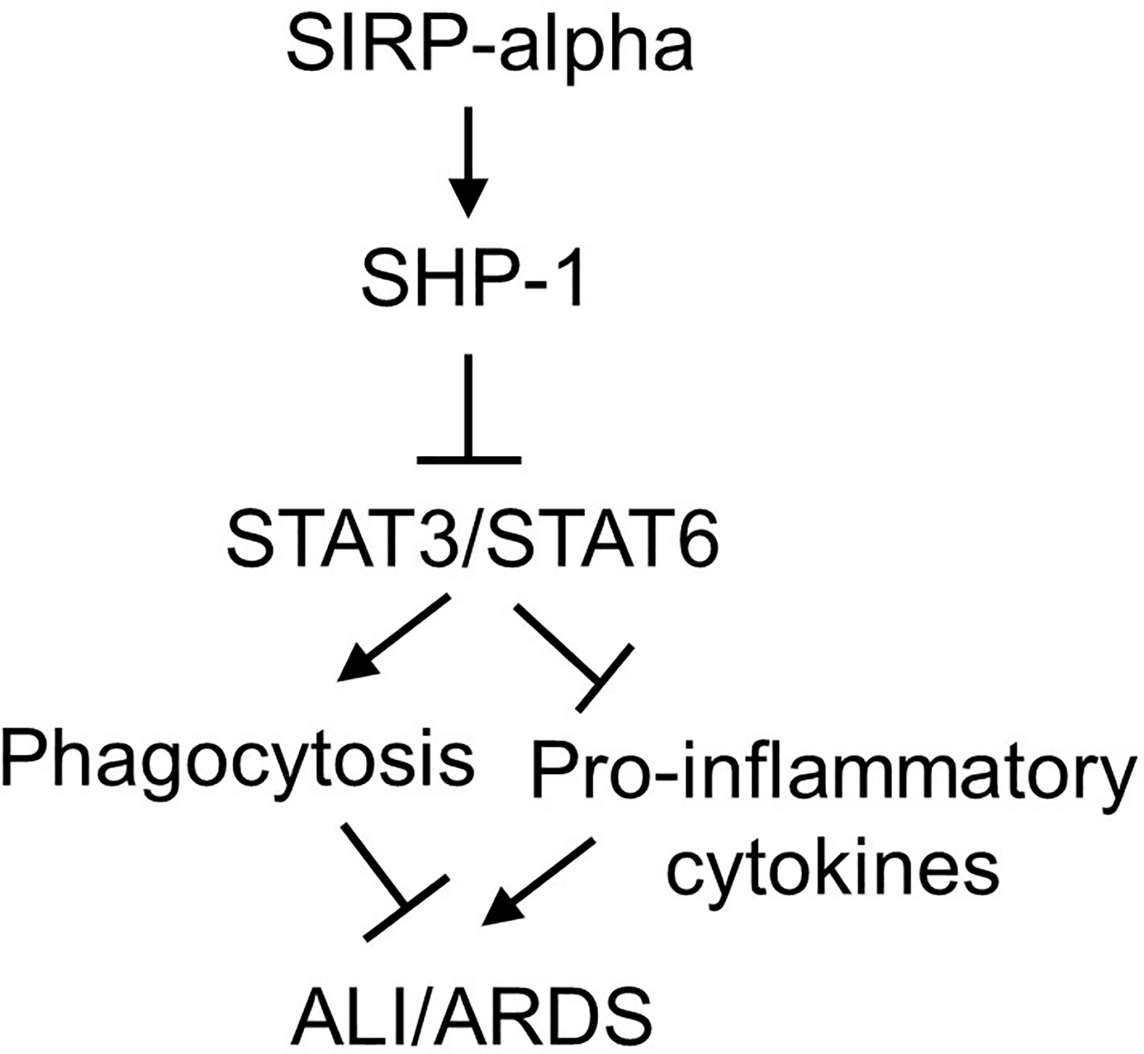
Schematic diagram of SIRP-alpha downstream signaling pathways in modulation of macrophages and development of ALI/ARDS. In LPS-induced ALI, the expression and release of soluble SIRP-alpha are increased, subsequently activates SHP-1, and suppresses activation of STAT3 and STAT6. Activation of STAT3 and STAT6 promotes macrophage phagocytosis, clearance of apoptotic and dead cells in the inflamed lung tissues, subsequently improving inflammation resolution and tissues repair in ALI/ARDS.

It was documented that SIRP-alpha activated downstream protein SHP-1 (6, 28). To further investigate the involvement of SHP-1 in the modulation of STAT3 and STAT6 activation, we treated WT and KO BMDMs with SHP-1 inhibitor, TPI-1. The results by immunostaining (**Figures 8D, E**) and flow cytometry analysis (**Figure 8F**) showed that TPI-1 largely increased activation of STAT3 and STAT6 in macrophages, indicating a suppressive role of SHP-1 in STAT3 and STAT6 signaling. Thus, lack of SIRP-alpha improved macrophage phagocytosis possibly through suppressing SHP-1 activation and subsequently inducing activation of STAT3 and STAT6 signaling pathways (**Figure 9**).

## Discussion

SIRP-alpha is well studied in cancer immunotherapy due to its potent immune suppressive role in macrophage phagocytosis. A body of evidence has showed that blocking SIRP-alpha signaling effectively inhibited tumor growth through enhanced macrophage phagocytosis (29). However, little is known about the expression and role of SIRP-alpha in animal model with ALI. To address this issue, we measured soluble SIRP-alpha protein content in ALI BAL and found that soluble SIRP-alpha expression level was significantly increased, compared to that in naïve mice. In addition, the soluble SIRP-alpha expression level in BAL was positively correlated to the expression levels of pro-inflammatory mediators in murine ALI. Thus, soluble SIRP-alpha in BAL could be considered as a novel biomarker in evaluation of murine ALI. In addition, we found that blocking SIRP-alpha activity in ALI BAL by neutralizing antibody significantly reduced macrophage activation and pro-inflammatory cytokine expression in macrophages, indicating the pro-inflammatory function of soluble SIRP-alpha in murine ALI. The results supported the previous report, in which over-expression of proteolytically cleaved SIRP-alpha induced activation of STAT1/NF-kappa B pathway *in vitro* (2). Therefore, we conclude for the first time that soluble SIRP-alpha was released into BAL of murine ALI under oxidative stress and had pro-inflammatory function.

To further clarify the biological function of SIRP-alpha in murine ALI, we created a SIRP-alpha KO mouse model with LPS-induced ALI. The results indicated that SIRP-alpha was expressed in both macrophages and neutrophils of WT mice. However, SIRP-alpha was unexpressed or expressed at low levels in SIRP-alpha^-/-^ and ^+/-^ mice. Lack of SIRP-alpha expression significantly attenuated the severity of murine ALI, confirming the pathogenic function of SIRP-alpha in murine ALI. The results were consistent with the previously reported results in other animal models, in which lack of SIRP-alpha protected mice from acute kidney injury and increasing insulin sensitivity in inflammation-mediated insulin resistance (17, 18).

Further analysis revealed that the attenuated severity of ALI in SIRP-alpha KO mice was associated with lower infiltrating Siglec-F(-) subtype of neutrophils and macrophages. Because neutrophils and macrophages are major sources of debilitating matrix metallopeptidase 2/9 (MMP2/9), ROS and other pro-inflammatory mediators (30, 31), the reduced infiltration of neutrophils and macrophages should contribute to the beneficial effects of SIRP-alpha deficiency in murine ALI.

However, it is unclear so far whether the reduced ratio of Siglec-F(-)/Siglec-F+ subtype macrophages and neutrophils contributed to the lower severity of ALI. According to our previous reports (25, 27), Siglec-F was co-expressed with CD206+ alternatively activated macrophages (M2 cells), we think that Siglec-F could be considered as an alternative M2 cell biomarker, and may play an immune regulatory role. The concept is supported by previous reports, in which Siglec protein can suppress chemotaxis of macrophages and neutrophils (32–35), that was evidenced by reduced adhesion and rolling of neutrophils and macrophages on endothelial cells in response to Siglec F expression. Thereby, we speculated that lack of SIRP-alpha expression attenuated murine ALI possibly through reducing the ratio of Siglec-F(-)/Siglec-F+ subtype macrophages and neutrophils in KO mice, subsequently suppressing the chemotaxis of macrophages and neutrophils *in vivo*. That will be further clarified in the future.

It was well documented that macrophage phagocytosis plays an important role in inflammation resolution (36). Our further *in vitro* study confirmed that SIRP-alpha deficiency in macrophages from KO mice or blocking SIRP-alpha activity on macrophage surface by neutralizing antibody effectively improved macrophage phagocytosis of apoptotic neutrophils, and significantly reducing production of pro-inflammatory cytokines and chemokines, such as TNF-alpha, IL-6 and RANTES. The effects were also observed in macrophages treated with BAL pre-neutralized by SIRP-alpha antibody, in which blockade of soluble SIRP-alpha in BAL significantly increased macrophage phagocytosis activity and reduced production of pro-inflammatory cytokines and chemokines. It should be noted that lack of SIRP-alpha on macrophage surface did not affect the effects of soluble SIRP-alpha on macrophages, because blocking soluble SIRP-alpha activity in BAL can effectively suppress macrophage phagocytosis and production of pro-inflammatory cytokines in KO BMDMs (data not shown). Therefore, membrane-bound and soluble SIRP-alpha both have pro-inflammatory properties on macrophages. Soluble SIRP-alpha may affect macrophage function through targeting unknown receptor on macrophages.

CD47 is a ligand of SIRP-alpha. In addition to the suppressive role of CD47 and SIRP-alpha interaction in tumor cell phagocytosis (29, 37), CD47 and SIRP-alpha interaction can promote trafficking of SIRP-alpha+CD103(-) dendritic cells into mediastinal and mesenteric lymph nodes and driving Th17 and Th2-biased responses. Additional reports showed that CD47/SIRP-alpha interaction participated in the development of ischemia reperfusion-induced acute kidney injury, colitis and allergic asthma (18, 20, 38). Thus, we speculate that CD47/SIRP-alpha interaction may be involved in the development of ALI. It warrants us to future investigate the role of CD47 in SIRP-alpha mediated murine ALI in the future.

According to the previous reports, SIRP-alpha activates downstream intracellular molecule SHP-1, leading to the suppression of macrophage activation and phagocytosis (39, 40); whereas activation of STAT3 (41, 42) and STAT6 (43, 44) signaling can promote macrophage phagocytosis. Thus, we speculate that lack of SIRP-alpha attenuated murine ALI possibly through suppressing SHP-1 and activating STAT3 and STAT6 signaling pathways. To address this issue, we treated macrophages with SHP-1, STAT3 and STAT6 inhibitors. The results revealed that lack of SIRP-alpha suppressed activation of SHP-1, and increased activation of STAT3 and STAT6. The results indicated the role of SIRP-alpha in activation of SHP-1 and suppression of STAT3 and STAT6 signaling. Thus, suppression of SHP-1 by inhibitor improved activation of STAT3 and STAT6. The results demonstrated the suppressive role of SHP-1 in activation of STAT3 and STAT6. According to the previous reports (45–47), STAT3 and STAT6 can induce anti-inflammatory responses and suppress transcription of pro-inflammatory mediators, we speculate that the increased STAT3 and STAT6 signaling in SIRP-alpha deficient macrophages contributed to the improved macrophage phagocytosis and suppressed production of pro-inflammatory mediators. Thereby, we conclude that lack of SIRP-alpha expression attenuated ALI possibly through activation of STAT3 and STAT6 signaling, and subsequently improving macrophage phagocytosis. SIRP-alpha exerts pro-inflammatory effects by activation of SHP-1 and inhibition of STAT3 and STAT6 signaling in macrophages (**Figure 9**).

However, it should be noted that LPS also increased macrophage phagocytosis and induced additive effects in conjunction with blockade of SIRP-alpha signaling in this study. The results were consistent with a previous report by Nepal S, et al, in which LPS treatment induced efferocytosis of apoptotic polymorphonuclear leukocytes (PMNs) by increasing the expression of anti-inflammatory IL-4 and tumor necrosis factor-stimulated gene-6 (TSG-6), subsequently activated STAT6 and the expression of growth arrest-specific 6 (Gas6) (48). The role of LPS in activation of STAT6 and its homologous STAT6(B) was also reported by another group (49). Thus, we explain that LPS improved macrophage phagocytosis predominantly through STAT3/STAT6 signaling, independent of SIRP-alpha in this study (**Figure 9**). We expect that LPS-induced improvement in macrophage phagocytosis should have beneficial feedback effects on inflammation resolution and tissue repair at later phase of inflammation, in together with the increased differentiation of anti-inflammatory regulatory T cells (50, 51).

Taken together, lack of SIRP-alpha expression attenuated the severity of murine ALI. The effects were mediated through improving STAT3 and STAT6 signaling, subsequently increasing macrophage phagocytosis. Thus, SIRP-alpha plays a pro-inflammatory role in murine ALI. Targeting SIRP-alpha by neutralizing antibody and molecular intervention would be a promising therapeutic approach in the treatment of ALI/ARDS.

## Data Availability Statement

The raw data supporting the conclusions of this article will be made available by the authors, without undue reservation.

## Ethics Statement

The animal study was reviewed and approved by Institutional Animal Care and Use Committee at Zhongshang Hospital Fudan University.

## Author Contributions

QS, LZ, LP, and DL participated in cell culture, Western blot and ELISA analysis. GC and ZC participated in the generation of hypothesis. ZJ participated in the generation of hypothesis, animal experiments, flow cytometry, data analysis and assembly, manuscript writing, revision, and was responsible for all direction of the work. All authors read and approved the final manuscript.

## Funding

This study was supported by grants from the Natural Science Foundation of Shanghai to ZJ (19ZR1409000), National Natural Science Foundation of China to ZC (81970023), and Doctoral Scientific Research Foundation of Henan Provincial People’s Hospital, Zhengzhou, Henan, China to DL (ZC20190166).

## Conflict of Interest

The authors declare that the research was conducted in the absence of any commercial or financial relationships that could be construed as a potential conflict of interest.

## Publisher’s Note

All claims expressed in this article are solely those of the authors and do not necessarily represent those of their affiliated organizations, or those of the publisher, the editors and the reviewers. Any product that may be evaluated in this article, or claim that may be made by its manufacturer, is not guaranteed or endorsed by the publisher.

## Abbreviations

ADAM10, a disintegrin and metalloproteinase domain-containing protein 10; ALI, murine acute lung injury; ARDS, acute respiratory distress syndrome; aSIRP, anti-SIRP-alpha antibody; BAL, bronchoalveolar lavage; BMDMs, bone marrow-derived macrophages; CD47, cluster of differentiation 47; Gas6, growth arrest-specific 6; GM-CSF, granulocyte-macrophage colony-stimulating factor; KO, knock-out; LPS, lipopolysaccharides; M2 cells, alternatively activated macrophages; MPs, macrophages; NPs, neutrophils; ROCK, Rho-associated protein kinase; ROS, reactive oxygen species; SHP-1, Src homology 2 domain-containing protein tyrosine phosphatase-1; SIRP-alpha, signal regulatory protein-alpha; SP-D and A, Surfactant protein-D and A; TSG-6, tumor necrosis factor-stimulated gene-6; TSP-1, thrombospondin-1; WT, wild-type.
